# Monovision by Implantation of Posterior Chamber Phakic Intraocular Lens with a Central Hole (Hole ICL) for Early Presbyopia

**DOI:** 10.1038/s41598-017-11539-9

**Published:** 2017-09-12

**Authors:** Kazutaka Kamiya, Masahide Takahashi, Natsumi Takahashi, Nobuyuki Shoji, Kimiya Shimizu

**Affiliations:** Department of Ophthalmology, University of Kitasato School of Medicine, Kanagawa, Japan

## Abstract

This study was aimed to evaluate visual performance at near to far distances in early presbyopic patients undergoing monovision by implantation of an ICL with a central hole (hole ICL). This pilot study comprised thirty-four eyes of 17 early presbyopic patients (age, 40 to 53 years) who underwent hole ICL implantation, and whose targeted refraction was set at emmetropia for the dominant eye, and at slight myopia (−0.5 to −1.0 diopters (D)) for the non-dominant eye. Corrected distance visual acuity was significantly improved, from −0.11 ± 0.07 preoperatively to −0.19 ± 0.09 logMAR postoperatively (p < 0.001, Wilcoxon signed-rank test). Uncorrected distance visual acuity was also significantly improved from 1.43 ± 0.35 preoperatively to −0.04 ± 0.18 logMAR postoperatively (p < 0.001). The mean binocular visual acuity was 0.01 logMAR or better at all distances (5.0, 3.0, 2.0, 1.0, 0.7, 0.5, and 0.3 m). All eyes were within ± 0.5 D of the targeted correction. All patients had within the normal range of near stereopsis. Neither cataract formation, significant intraocular pressure rise, nor vision-threatening complications occurred. Monovision by hole ICL implantation provided good binocular vision at near to far distances, without developing cataract, suggesting its feasibility as a new surgical presbyopic approach for early presbyopia.

## Introduction

The posterior chamber phakic intraocular lens (Visian ICL, STAAR Surgical, Nidau, Switzerland) has been reported to provide the long-term effective outcomes for moderate to high ametropia^1–4^. A new ICL with an artificial central hole (hole ICL, KS-AP^TM^; STAAR Surgical) has been developed in order to avoid the occurrence of pupillary block even without preoperative laser iridotomies, and to minimize the rate of developing cataract especially in older patients^5–7^.

Monovision technique is one of viable options for the management of presbyopia where the one eye is corrected for distance vision and the other for near. However, this technique was not usually utilized when performing conventional ICL (without a central hole) implantation, because of the higher rate of cataract formation in these presbyopic subjects^8–10^. Considering that hole ICL may considerably decrease the risk of cataract formation in such patients, presumably resulting from improving the circulation of the aqueous humour^11^, it is possible that monovision by hole ICL implantation contributes to obtaining good binocular visual performance from near to far distances in early presbyopic subjects, without developing cataract. The aim of the current study is to prospectively evaluate the safety, efficacy, and predictability of monovision by hole ICL implantation for the correction of moderate to high ametropia in early presbyopic subjects, with special attention to binocular visual performance at near to far distances.

## Results

### Study Population

The preoperative and postoperative demographics of the study population are summarized in Table 1. There were no intraoperative complications, and all surgeries were uneventful. No eyes were lost during the 6-month observation period in this series.

**Table 1:** Preoperative demographics of the study population undergoing hole implantable collamer lens (hole ICL) implantation.

Characteristic	Mean ± Standard deviation
Age (years)	46.1 ± 4.2 years (range, 40 to 53 years)
Gender (Male: Female)	8: 9
Manifest spherical equivalent (D)	−8.67 ± 4.35 D (range, −2.25 to −18.25 D)
Manifest cylinder (D)	0.80 ± 0.76 D (range, 0.00 to 3.00 D)
LogMAR UDVA	1.43 ± 0.35 (range, 0.52 to 2.00)
LogMAR CDVA	−0.11 ± 0.07 (range, −0.18 to 0.00)
Targeted refraction in the dominant eye (D)	−0.08 ± 0.09 D (range, −0.25 to −0.00 D)
Targeted refraction in the non-dominant eye (D)	−0.66 ± 0.18 D (range, −0.455 to −1.00 D)
White-to-white distance (mm)	11.7 ± 0.4 mm (range, 11.0 to 12.6 mm)
Anterior chamber depth (mm)	3.14 ± 0.28 mm (range, 2.82 to 3.74 mm)

D = diopter, logMAR = logarithm of the minimal angle of resolution, UDVA = uncorrected distance visual acuity, CDVA = corrected distance visual acuity.

### Safety Outcomes

**Figure 1:**
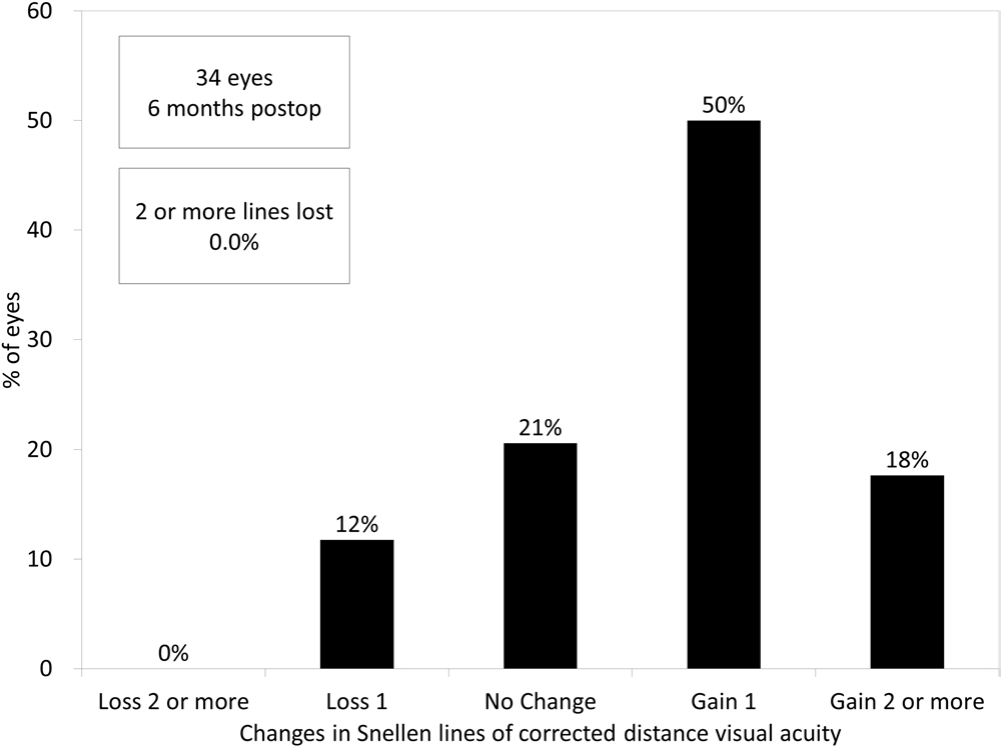
Changes in corrected distance visual acuity (CDVA) after hole implantable collamer lens (Hole ICL) implantation.

CDVA was significantly improved, from −0.11 ± 0.07 preoperatively to −0.19 ± 0.09 logMAR postoperatively (p < 0.001, Wilcoxon signed-rank test). Seven eyes (21%) showed no change in CDVA, 17 eyes (50%) gained 1 line, 6 eyes (18%) gained 2 lines, and 4 eyes (12%) lost 1 line, 6 months postoperatively (Fig. 1). Although 4 eyes lost 1 line of CDVA, all these eyes had a CDVA of 20/20 or more.

### Efficacy Outcomes at Near to Far Distances

**Figure 2:**
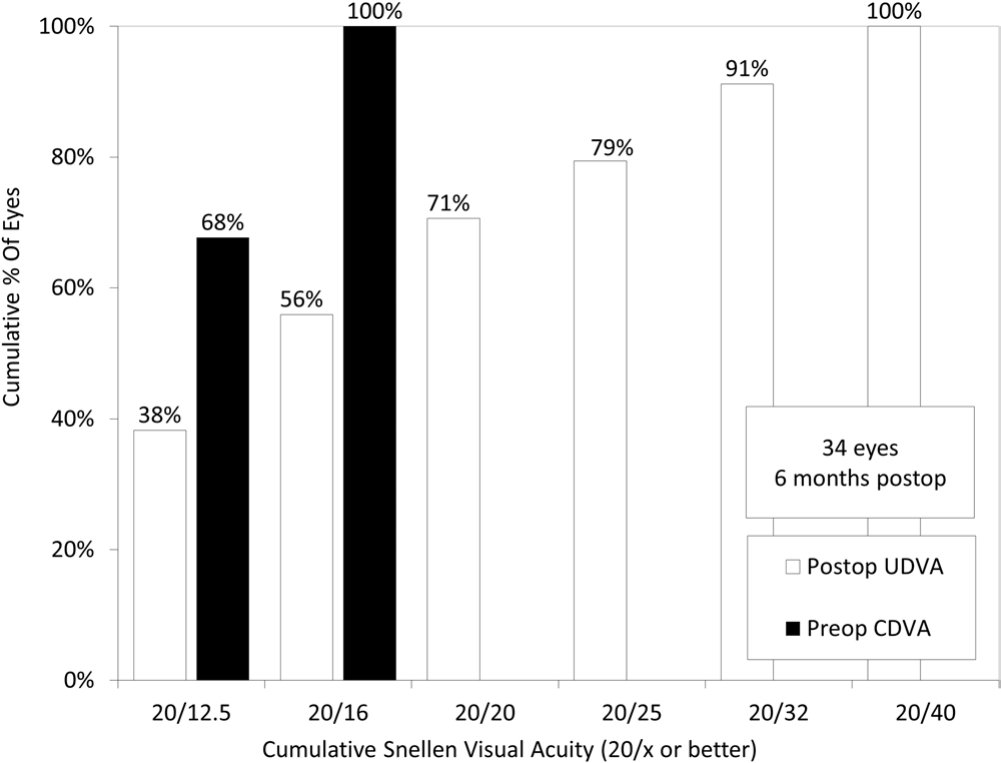
Cumulative percentages of eyes attaining specified cumulative levels of uncorrected distance visual acuity (UDVA) after hole implantable collamer lens (hole ICL) implantation.

**Figure 3:**
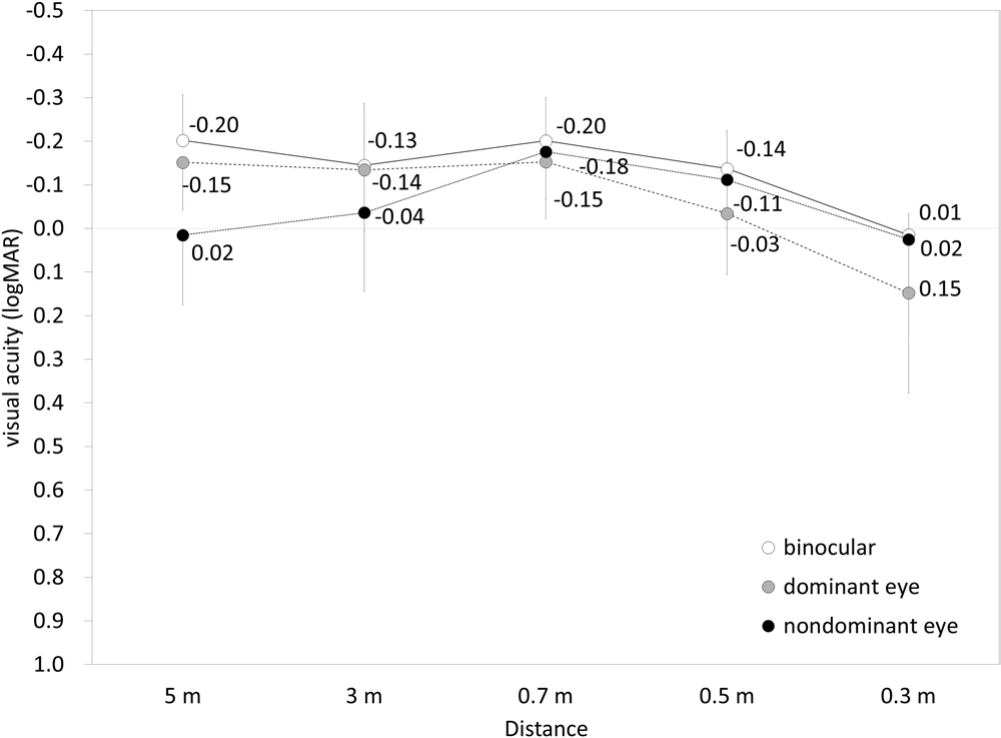
Uncorrected visual acuity at near to far distances after hole implantable collamer lens (hole ICL) implantation.

Near monocular and binocular visual acuities (at 0.3 m) when correcting far vision preoperatively were 0.17 ± 0.23 and 0.10 ± 0.19 logMAR, respectively. UDVA was also significantly improved from 1.43 ± 0.35 preoperatively to −0.04 ± 0.18 logMAR postoperatively (p < 0.001, Wilcoxon signed-rank test). The cumulative percentages of eyes attaining specified cumulative levels of UDVA and the postoperative binocular uncorrected visual acuity at 5.0, 3.0, 2.0, 1.0, 0.7, 0.5, and 0.3 m distances are shown in Figs 2 and 3, respectively. The mean binocular visual acuity was 0.01 logMAR or better at all distances. Sixteen of 17 patients (94%) had simultaneous binocular acuities of 20/25 or better at far and J2 or better at near.

### Predictability

**Figure 4:**
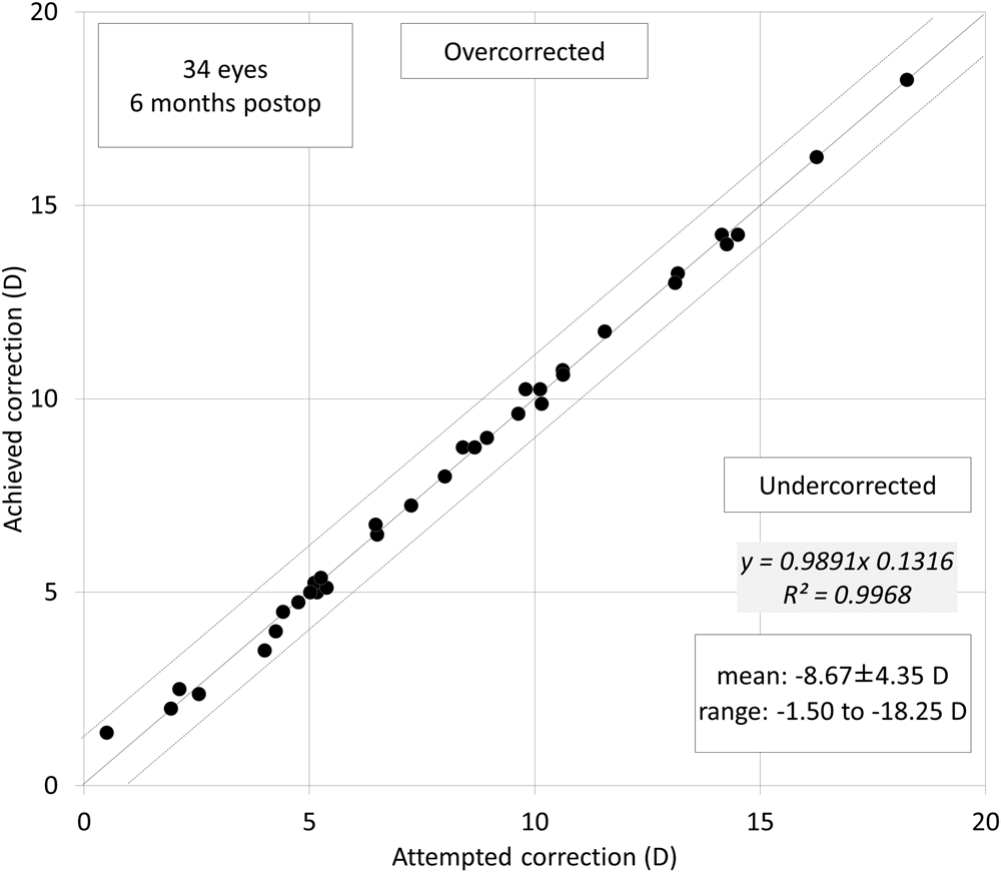
A scatter plot of attempted vs. achieved correction (spherical equivalent) after hole implantable collamer lens (hole ICL) implantation.

A scatter plot of the attempted versus the achieved correction is shown in Fig. 4. The achieved refraction was −0.08 ± 0.17 diopters (D) in the dominant eye, and −0.65 ± 0.29 D in the non-dominant eye. All eyes were within ± 0.5 D of the targeted correction.

### Near Stereopsis, Contrast Sensitivity, and Patient Satisfaction

**Figure 5:**
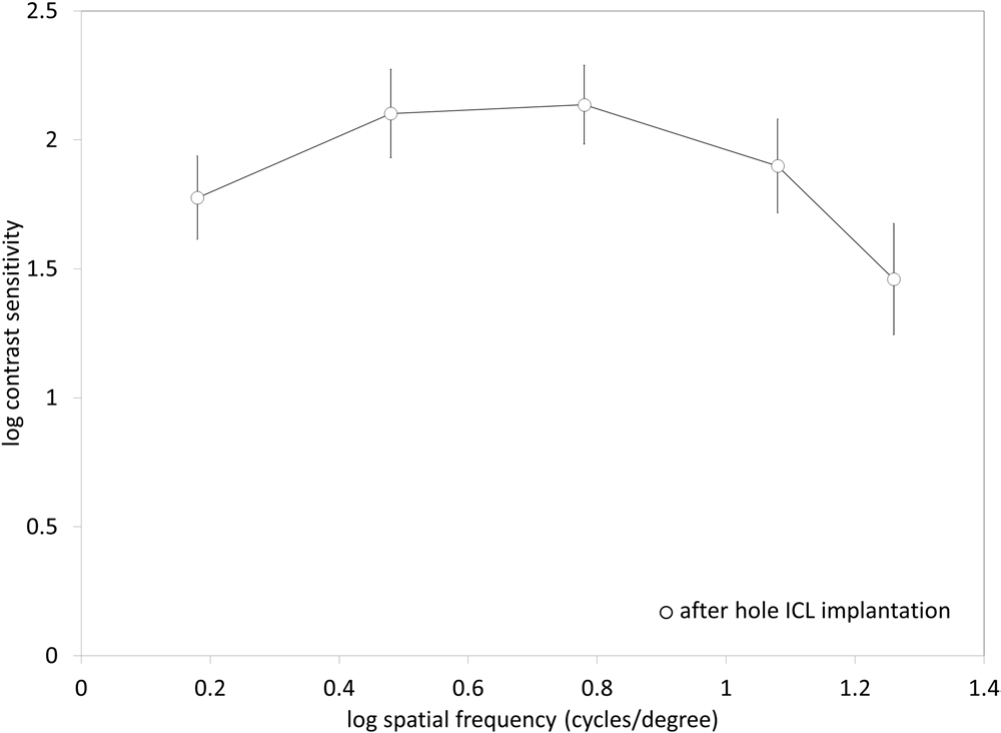
Contrast sensitivity function after hole implantable collamer lens (hole ICL) implantation.

All patients had within normal range of near stereopsis (40 seconds of arc, 15 eyes, 50 seconds of arc, 1 eye, and 60 seconds of arc, 1 eye) without refractive correction. The postoperative contrast sensitivity was shown in Fig. 5. All eyes had within normal range of contrast sensitivity function. The postoperative satisfaction score was 9.1 ± 0.8 (range: 8 to 10). All patients have satisfied with overall visual performance after hole ICL implantation.

### Adverse events

Neither cataract formation, intraocular pressure rise (≥22 mmHg), pigment dispersion glaucoma, pupillary block, nor any other severe complications, occurred in any case during the 6-month follow-up period.

## Discussion

In the current study, our pilot study showed that binocular uncorrected visual acuity at all distances was overall good even in early presbyopic patients, and that no definite complications were found during the observation period, suggesting its feasibility as a surgical correction for eyes with moderate to high ametropia and early presbyopia. To the best of our knowledge, this is the first study to assess the monovision technique by the use of hole ICLs for early presbyopic subjects. We also confirmed that monovision by hole ICL implantation was overall good in terms of the safety, efficacy, and predictability for the correction of moderate to high ametropia, which was in line with previous studies on ICL impantation^1–7^. We measured preoperative CDVA with spectacle correction in the present study. The retinal magnification with spectacle correction is smaller than that with ICL correction especially in high myopic eyes, because the location of the ICL is closer to a nodal point. Accordingly, the improvement of CDVA may be partly explained by less magnification of the retinal image in the study population.

Other monovision techniques include spectacle, contact lens, corneal inlay, LASIK, and refractive lens exchange. When presbyopic patients having moderate to high ametropia wish to undergo surgical correction, corneal inlay and presbyopic LASIK may induce a large amount of higher-order aberrations, and thus decrease contrast sensitivity function especially in mesopic conditions. Refractive lens exchange may lose the accommodation of the crystalline lens, and increase the possible risk of retinal detachment. Although conventional ICL implantation may increase the rate of cataract formation in older patients^8–10^, hole ICL implantation has been reported to considerably reduce the risk of cataract formation, possibly because of the improvement of the circulation of the aqueous humour to the anterior surface of the crystalline lens^5–7^. Therefore, we selected hole ICL implantation for early presbyopic patients with moderate to high ametropia in the current study. Neither asymptomatic nor symptomatic cataract formation occurred in this series. However, it should be noted that there are some possibilities of glare and halo at night especially in eyes with larger pupil and those of the postoperative rotation in toric ICL-implanted eyes, and that LASIK takes less time and less expensive than ICL implantation.

One limitation to the present study is that the sample size was small with a 6-month follow-up, because this is a pilot study to explore the safety and efficacy of this new presbyopic approach. Accordingly, we awaits further investigations with a large number of patients on the long-term incidence of cataract formation as well as other complications in this study population. Another limitation is that we did not assess contrast sensitivity function under mesopic and glare conditions in these eyes. It will be helpful for understanding more detailed visual performance after hole ICL implantation.

In conclusion, our findings showed that monovision by hole ICL implantation is beneficial for acquiring overall good binocular visual performance at all distances in early presbyopic subjects without developing cataract. It is suggested that monovision by hole ICL implantation is helpful for obtaining better visual performance at near to far distances in such presbyopic subjects.

## Methods

### Study Population

This study comprised 34 eyes of 17 patients (8 men and 9 women), who underwent hole ICL implantation for the correction of moderate to high myopia, and whose age was 40 years or more with subjective symptoms of near visual disturbance when correcting far vision. The inclusion criteria for this surgical technique were as follows: unsatisfactory correction with spectacles or contact lenses, stable refraction for at least 6 months, −2.00 to −18.50 D of myopia with astigmatism of 3 D or less, anterior chamber depth ≥2.8 mm, endothelial cell density ≥1800 cells/mm^2^, no history of ocular surgery, corneal degeneration, cataract, glaucoma, uveitis, or diabetic retinopathy. Keratoconic eyes were excluded from the study by the use of the keratoconus screening test of Placido disk videokeratography (TMS-2, Tomey, Nagoya, Japan). We selected non-toric ICL in 26 eyes (76%) with the manifest cylinder of 1.25 D or less, or toric ICL in 8 eyes (34%) with that of 1.5 D or more, respectively. Preoperatively and 6 months postoperatively, we evaluated the following parameters: the logarithm of the minimal angle of resolution (logMAR) of monocular distance visual acuity (UDVA) binocular uncorrected visual acuity at 5.0, 3.0, 2.0, 1.0, 0.7, 0.5, and 0.3-m distances (only postoperatively), logMAR corrected distance visual acuity (CDVA), and manifest refraction (spherical equivalent and cylinder), in addition to the usual slit-lamp biomicroscopic and funduscopic examinations. Visual acuity measurement was performed under photopic conditions (250 lux) using a trial frame and a Snellen chart with Japanese letters at a distance of 5 m. Binocular visual acuity measurements were performed at 5.0, 3.0, 2.0, 1.0, 0.7, 0.5, and 0.3 m using an all-distance vision tester (AS-15, Kowa, Tokyo, Japan). This device measures equivalent visual acuity from far to near distances by placing a spherical lens and various visual targets at proper distances along the visual axis. Refraction was measured using an automated refractometer (ARK-700A, Nidek, Gamagori, Japan), and the results were used as a starting point for a full manifest refraction. We also assessed near stereopsis, contrast sensitivity function, and patient satisfaction, 6 months postoperatively. Near stereopsis was measured using the Titmus stereo test at 40 cm without refractive correction. Contrast sensitivity function was measured by a contrast sensitivity unit (VCTS-6500, Vistech) under photopic conditions (500 lux). The test was performed with best spectacle correction at 2.5 m. The patient satisfaction for overall visual performance was assessed according to the visual analog scale in a range from 0 (very dissatisfied) to 10 (very satisfied) by one of the authors who did not participate in the overall treatment or follow-up of the patients in the study. All examinations were performed by experienced optometrists, who were masked to the clinical condition of the subjects. The study was approved by the Institutional Review Board at Kitasato University School of Medicine, and followed the tenets of the Declaration of Helsinki. Informed consent was obtained from all patients after explanation of the nature and possible consequences of the study.

### Lens Power and Size Calculation

Hole ICL power calculation was performed by the manufacturer’s software (STAAR Surgical). We selected emmetropia in the dominant eye and −0.5 to −1.0 D in the non-dominant eye as the target refraction, which was individually determined with visual simulation with contact lenses wearing in each patient, based on patient age and patient preference for vision. The size of the ICL was also selected by the manufacturer’s nomogram based on the horizontal white to white distance and the anterior chamber depth.

### Surgical Procedure

The patients were given dilating and cycloplegic agents 3 times before surgery. After topical anesthesia, hole ICL was slowly pushed into the anterior chamber through a 3-mm temporal corneal incision, following placement of a viscosurgical device into the anterior chamber. The haptics of the ICL was fixated into the ciliary sulcus using the manipulator. The remaining viscosurgical device was fully replaced with balanced salt solution. A miotic agent was administrated. Postoperatively, we used topical steroidal and antibiotic medications 4 times daily for 2 weeks, the dose being reduced gradually thereafter.

### Statistical Analysis

All statistical analyses were performed using a commercially available statistical software (Ekuseru-Toukei 2015, Social Survey Research Information Co, Ltd., Tokyo, Japan). Only one eye from each patient was selected randomly when comparing monocular preoperative and postoperative visual acuities for statistical analysis. The Wilcoxon signed-rank test was used for statistical analysis to compare the pre- and post-treated data. The results are expressed as mean ± standard deviation, and a value of p < 0.05 was considered statistically significant.
